# Reliability, construct and concurrent validity of a smartphone-based cognition test in multiple sclerosis

**DOI:** 10.1177/13524585211018103

**Published:** 2021-05-26

**Authors:** KH Lam, P van Oirschot, B den Teuling, HE Hulst, BA de Jong, BMJ Uitdehaag, V de Groot, J Killestein

**Affiliations:** Department of Neurology, Amsterdam University Medical Centers, Vrije Universiteit Amsterdam, MS Center Amsterdam, Amsterdam Neuroscience, Amsterdam, The Netherlands; Orikami Digital Health Products, Nijmegen, The Netherlands; Orikami Digital Health Products, Nijmegen, The Netherlands; Department of Anatomy and Neurosciences, Amsterdam University Medical Centers, Vrije Universiteit Amsterdam, MS Center Amsterdam, Amsterdam Neuroscience, Amsterdam, The Netherlands; Department of Neurology, Amsterdam University Medical Centers, Vrije Universiteit Amsterdam, MS Center Amsterdam, Amsterdam Neuroscience, Amsterdam, The Netherlands; Department of Neurology, Amsterdam University Medical Centers, Vrije Universiteit Amsterdam, MS Center Amsterdam, Amsterdam Neuroscience, Amsterdam, The Netherlands; Department of Rehabilitation Medicine, Amsterdam University Medical Centers, Vrije Universiteit Amsterdam, MS Center Amsterdam, Amsterdam Neuroscience, Amsterdam, The Netherlands; Department of Neurology, Amsterdam University Medical Centers, Vrije Universiteit Amsterdam, MS Center Amsterdam, Amsterdam Neuroscience, Amsterdam, The Netherlands

**Keywords:** Multiple sclerosis, cognition, digital biomarker, smartphone, ecological momentary assessment

## Abstract

**Background:**

Early detection and monitoring of cognitive dysfunction in multiple sclerosis (MS) may be enabled with smartphone-adapted tests that allow frequent measurements in the everyday environment.

**Objectives:**

The aim of this study was to determine the reliability, construct and concurrent validity of a smartphone-adapted Symbol Digit Modalities Test (sSDMT).

**Methods:**

During a 28-day follow-up, 102 patients with MS and 24 healthy controls (HC) used the MS sherpa^®^ app to perform the sSDMT every 3 days on their own smartphone. Patients performed the Brief International Cognitive Assessment for MS at baseline. Test–retest reliability (intraclass correlation coefficients, ICC), construct validity (group analyses between cognitively impaired (CI), cognitively preserved (CP) and HC for differences) and concurrent validity (correlation coefficients) were assessed.

**Results:**

Patients with MS and HC completed an average of 23.2 (*SD* = 10.0) and 18.3 (*SD* = 10.2) sSDMT, respectively. sSDMT demonstrated high test–retest reliability (ICCs > 0.8) with a smallest detectable change of 7 points. sSDMT scores were different between CI patients, CP patients and HC (all *p*s < 0.05). sSDMT correlated modestly with the clinical SDMT (highest *r* = 0.690), verbal (highest *r* *=* 0.516) and visuospatial memory (highest *r* *=* 0.599).

**Conclusion:**

Self-administered smartphone-adapted SDMT scores were reliable and different between patients who were CI, CP and HC and demonstrated concurrent validity in assessing information processing speed.

## Introduction

Cognitive impairment is common in patients with MS and has a disabling impact on daily living.^1^ Cognitive deficits are associated with disability progression and relapses^2^ and can be present early in the disease or even prior to clinical or radiological disease onset.^3^ Therefore, assessment of cognition as a marker for disease progression and disease activity may lead to more timely and targeted treatment interventions.^4^ Information processing speed is the most affected cognitive domain and also an indicator for the overall impact of cognitive functioning over time in MS.^5^ The assessment of information processing speed has therefore been recommended for the screening and monitoring of cognitive functioning.^6^

We hypothesise that smartphone-based assessment of information processing speed in the everyday environment better reflects real-life cognitive functioning than periodic momentary neuropsychological assessment in the clinical setting. Assessment of cognition in MS through wearable devices, such as tablets and smartphones, has been studied previously.^7–13^ These mostly instrumented versions of the Symbol Digit Modalities Test (SDMT) could differentiate patients with MS from healthy controls (HC) and were found to be reliable and valid for measuring information processing speed.^8,13^ However, digital monitoring tools for cognition are not yet employed in clinical practice due to different challenges including the lack of standardisation.^14^ Here, we expand on previous work on smartphone-based assessment of information processing speed by analysing the clinimetric properties of a smartphone-adapted Symbol Digit Modalities Test (sSDMT) to provide a basis towards clinical implementation.

### Objective

The aim of this study was to determine the reliability, construct and concurrent validity of a smartphone-adapted SDMT to clinical outcomes in MS with regard to the optimal frequency and time of assessment.

## Patients and methods

### Participants and study design

This study is part of an ongoing cohort study at Amsterdam UMC, location VU University Medical Centre. Following a baseline clinical study visit, participants installed and used the MS sherpa® app on their own smartphones in the everyday environment. Participants were consecutively included from August 2018 until a sample size of approximately 100 patients and 25 HC was reached in December 2019. Eligibility criteria included age between 18 and 65 years, use of a smartphone with Android (5.0 or higher) or iOS (10 or higher), no presence of visual or upper extremity deficits affecting regular smartphone use and no mood or sleep disorder impacting daily living based on medical history taking by a screening physician, and additionally, for patients, a definite diagnosis of MS and baseline Expanded Disability Status Scale (EDSS) score below 7.5. The study received full ethical approval (reference 2017.576) and conformed to legislation regarding data privacy and medical devices.

### Clinical assessments

At baseline, the following clinical assessments were performed in patients with MS: severity of clinical disability was quantified with the EDSS,^15^ manual dexterity was assessed with the Nine-Hole Peg Test (NHPT) and the Arm function in MS Questionnaire (AMSQ),^16,17^ and cognitive function was measured with the Brief International Cognitive Assessment for Multiple Sclerosis (BICAMS).^18^ The BICAMS consisted of the clinical Symbol Digit Modalities Test (cSDMT, oral version),^4^ the Dutch version of the California Verbal Learning Test (CVLT, immediate recall)^19^ and the Brief Visuospatial Memory Test-Revised (BVMT-R, immediate recall).^20^

### Smartphone assessments

**Figure 1.**
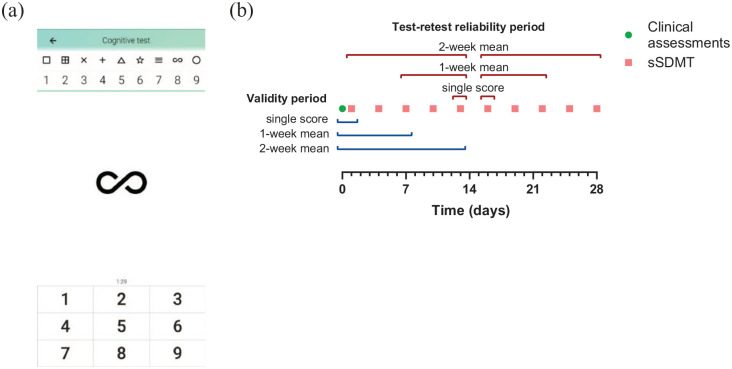
(a) Interface of the MS sherpa® sSDMT® 2017–2021 Orikami Digital Health Products. All rights reserved. (b) Schematic overview of the clinical (green dot) and smartphone (red squares) assessments. The red brackets represent the periods of data aggregation used for the test–retest reliability analysis. The blue brackets represent the periods of data aggregation used for the validity analysis. Abbreviations: sSDMT, smartphone Symbol Digit Modalities Test.

MS sherpa (Orikami Digital Health Products, Nijmegen)^21^ is a software as a medical device intended to monitor the presence and progression of symptoms related to MS. MS sherpa is a system consisting of a smartphone app (supported on Android and iOS) for data collection and data presentation, a cloud service for data storage, analysis algorithms and a clinician or research dashboard for user management and data presentation. The product is commercially available. More information can be found on the MS sherpa website.^21^ The app was installed on the own smartphones of the participants during the baseline visit and was used during a follow-up period of 4 weeks. MS sherpa includes a smartphone adaptation of the SDMT to assess information processing speed (see Figure 1(a)). The sSDMT was self-administered and performed by tapping the digits corresponding to each shown symbol on the smartphone screen. The number of correct responses after a 90-second trial is scored by the app. The symbol–digit combination is randomised in each trial. During the follow-up period, the sSDMT was assessed every 3 days during the morning (between 06:00 and 12:00) and in the evening (between 18:00 and 00:00). Push notifications were sent at 10:00 and 18:00 as reminders when a sSDMT task was scheduled.

### Statistical analysis

The statistical analysis was performed using SPSS version 26. Categorical data were summarised by numbers and percentages. Numerical data were summarised by the mean and standard deviation when normally distributed, otherwise the median and interquartile range were used. The *p*-values < 0.05 were considered statistically significant.

Test–retest reliability of the sSDMT scores was assessed by the calculation of the intraclass correlation coefficients (ICC). Single scores, 1- and 2-week mean scores (see Figure 1(b)) split between morning and evening assessments were analysed to investigate which period and time of the day of sSDMTs yielded the highest reliability estimates. A two-way mixed effects model on absolute agreement for single measurements was used. An ICC estimate cut-off of ⩾ 0.70 was used to indicate sufficient reliability.^22,23^ The smallest detectable change (SDC) was calculated from the ICC and standard error of measurement (SEM): $SDC=1.96\times \sqrt{2}\times SEM$ , where $SEM=SD\times \sqrt{1-ICC_{agreement}}$ . Bland–Altman plots were constructed to assess the systematic error (mean difference) and the 95% limits of agreement (mean difference ± 1.96 SD) between sSDMT test and retest scores.^24^

Construct validity of the sSDMT was analysed by assessing the ability of the sSDMT to distinguish HC and patients with MS who were classified as cognitively impaired (CI) and cognitively preserved (CP) according to the cSDMT cut-off score of 49.^25^ Group differences were analysed with Mann–Whitney U tests, and receiver operating characteristic (ROC) curves were analysed by plotting the sensitivity against the 1-specificity. The corresponding area under the ROC curve (AUC) values were reported with 95% confidence intervals.

Concurrent validity of the sSDMT to measure information processing speed was assessed by the calculation of Pearson’s correlation coefficient between the sSDMT and the clinical SDMT as reference measure. In addition, Pearson’s and Spearman’s correlation coefficients were calculated between the sSDMT and constructs of cognition (CVLT and BVMT-R) and overall disability (EDSS), respectively. Single score, 1- and 2-week mean scores (see Figure 1(b)) split between morning and evening were analysed to investigate which period and time of the day of sSDMTs yielded the highest correlation coefficients. Correlation coefficient sizes of < 0.3, 0.3–0.6 and > 0.6 were considered low, moderate and strong, respectively.^22^ Since the sSDMT was assessed by tapping the numbers on the own smartphone and compared to the oral clinical SDMT, the feasibility of the sSDMT in patients with MS was investigated. This was done with linear regression analysis to investigate whether the relation between smartphone and clinical SDMT was significantly confounded (i.e. ⩾ 10% change in regression coefficient) by age, sex, education, arm function (AMSQ and NHPT), severity of disability (EDSS) or size of the smartphone screen.

## Results

In total, 144 people were screened for eligibility of which 18 were excluded (no conventional use of smartphone, *n* = 6; age above 65 years, *n* = 5; no diagnosis of MS, *n* = 4; corneal dystrophy, *n* = 1; depression and sleeping disorder, *n* = 1; severe tremor, *n* = 1). Therefore, 102 patients with MS and 24 HC were included in the study. However, 11 patients with MS were removed from the analysis due to a software bug that slowed the performance of the sSDMT. Demographical and clinical characteristics of the remaining patients and HC at baseline are summarised in Table 1. During the 28-day follow-up, 92 patients completed a total of 2135 sSDMTs and 24 HC completed 439 sSDMTs. On average, each patient and HC completed 23.2 (*SD* = 10.0) and 18.3 (*SD* = 10.2) sSDMTs, respectively. Meanwhile, 7 patients (7.6%) and 6 HC (25.0%) had performed less than 15 (75% of the scheduled 20) smartphone cognition tests.

**Table 1.** Baseline demographical, clinical and smartphone characteristics.

	Patients with MS (*n* = 92)	HC (*n* = 24)	*p*-value
Age, years, mean (SD)	46.9 (10.1)	42.4 (15.1)	0.175^a^
Sex, *n* (%)			0.080^b^
Female	68 (73.9)	13 (54.2)	
Male	24 (26.1)	11 (45.8)	
Level of education^c^, *n* (%)			0.445^b^
Low	3 (3.3)	0 (0.0)	
Middle	29 (31.5)	5 (20.8)	
High	60 (65.2)	19 (79.2)	
MS type, *n* (%)			n/a
PPMS	11 (12.0)		
SPMS	26 (28.3)		
RRMS	55 (59.8)		
Disease duration, years, median (IQR)			n/a
Since onset	10.9 (5.3–18.3)		
Since diagnosis	6.3 (3.1–14.1)		
EDSS, median (range)	3.5 (1.5–7.0)		n/a
Clinical SDMT, mean (SD)	55.0 (10.2)		n/a
CI^d^, *n* (%)	23 (25.0)		
CP^d^, *n* (%)	69 (75.0)		
Smartphone operating system, *n* (%)			0.160^b^
Android	61 (66.3)	12 (50.0)	
iOS	31 (33.7)	12 (50.0)	
Smartphone screen size, median (IQR)			
Height, pixels	1920.0 (1334.0–2220.0)	1920.0 (1334.0–2214.0)	0.590^e^
Width, pixels	1080.0 (720.0–1080.0)	1080.0 (750.0–1080.8)	0.099^e^

MS: multiple sclerosis; PPMS: primary progressive multiple sclerosis; SPMS: secondary progressive multiple sclerosis; RRMS: relapsing–remitting multiple sclerosis; EDSS: Expanded Disability Status Scale; SDMT: Symbol Digit Modalities Test.

a Independent samples *t*-test.

b Fisher’s exact test.

c Level of education was stratified as low (primary school), average (low or medium level secondary school) and high (highest level secondary school, college degree, and/or university degree).^26^

d Cut-off value of 49 points.^25^

e Mann–Whitney U test.

### Test–retest reliability

**Figure 2.**
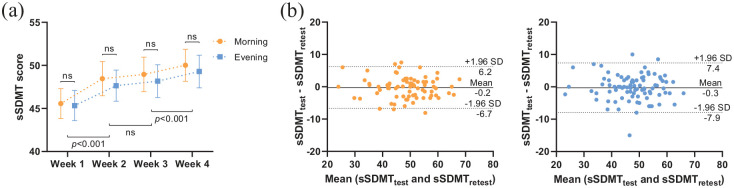
(a) Line graph (mean with 95% CI) of the sSDMT scores averaged per week during the follow-up period. Dependent-samples *t*-tests. (b) Bland–Altman plots of the sSDMT test and retest period (1-week averages) during the morning (orange) and evening (blue). The solid lines represent the mean difference, and the dashed lines represent the 95% limits of agreement. Abbreviations: sSDMT, smartphone Symbol Digit Modalities Test.

The sSDMT scores averaged per week are shown in Figure 2(a). sSDMTs performed during the morning were systematically higher than scores obtained during the evening, with a mean difference of 0.75 points. The weekly averaged sSDMT scores gradually increased during the follow-up period. An average increase in 4.1 and 3.7 points for the morning and evening scores, respectively, was observed between the first and last week, most likely due to practice effects. The results of the test–retest reliability are summarised in Table 2. Reliability estimates of the sSDMT were high (ICCs > 0.80). The reliability was highest for 1-week mean morning scores. Using this 1-week mean score, a score change of 6.7 points or more can be distinguished as a change beyond measurement error (i.e. the SDC). The Bland–Altman plot for the differences between the test (i.e. Week 2 mean scores) and the retest (i.e. Week 3 mean scores) plotted against the mean of the two periods is shown in Figure 2(b). The systematic difference between the test and retest was nearly 0. The limit of agreement was ± 6.4 and ± 7.6 points for the morning and evening sSDMT scores, respectively.

**Table 2.** Test–retest reliability and the SDC of the smartphone SDMT.

	Tests, *n* (mean ± SD)	Retests, *n* (mean ± SD)	*n*	ICC	SEM	SDC
Smartphone SDMT_M_						
Single score	1.0 ± 0.0	1.0 ± 0.0	87	0.882	3.09	8.58
1-week mean	2.4 ± 1.5	2.5 ± 1.7	78	0.934	2.33	6.47
2-week mean	5.8 ± 2.8	5.4 ± 3.1	87	0.918	2.47	6.86
Smartphone SDMT_E_						
Single score	1.0 ± 0.0	1.0 ± 0.0	89	0.875	3.12	8.66
1-week mean	2.3 ± 1.3	2.3 ± 1.1	88	0.900	2.70	7.49
2-week mean	5.6 ± 2.3	5.2 ± 2.3	89	0.914	2.50	6.92

ICC: intraclass correlation coefficient; SEM: standard error of measurement; SDC: smallest detectable change; SDMT_M_: smartphone Symbol Digit Modalities Test morning score; SDMT_E_: smartphone Symbol Digit Modalities Test evening score.

### Construct validity

**Figure 3.**
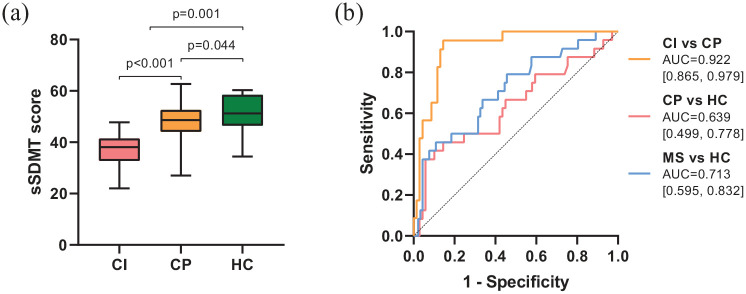
(a) Box-plot of the sSDMT scores between CI patients, CP patients and HC. Mann–Whitney U tests. (b) ROC curves showing the sensitivity and 1-specificity in distinguishing CI patients, CP patients and HC with the sSDMT. AUC-values and 95% confidence intervals (in brackets) are reported. Abbreviations: sSDMT, smartphone Symbol Digit Modalities Test; CI, patients with MS classified as cognitively impaired; CP, patients with MS classified as cognitively preserved; HC, healthy controls; ROC curve, receiver operating characteristic curve; AUC, area under the curve.

Group differences in sSDMT scores between patients with MS divided between CI and CP patients, and HC are shown in Figure 3(a). Patients with MS had lower median sSDMT scores compared to HC, *p* = 0.001. The ROC curve analyses are shown in Figure 3(b). Using sSDMT scores, CI patients could be distinguished from CP patients with an AUC-value of 0.922 (*p* < 0.001). AUC-values for classifying HC from patients with MS or CP patients were 0.713 (*p* = 0.001) and 0.639 (*p* = 0.044), respectively.

### Concurrent validity

**Figure 4.**
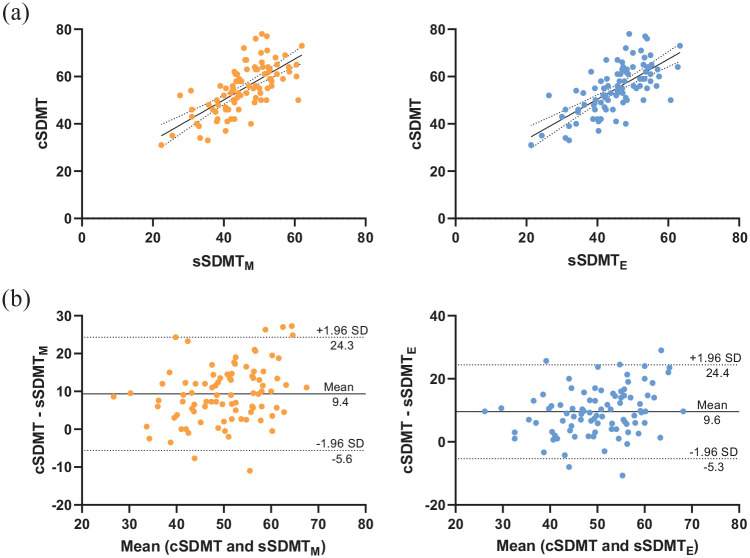
(a) Scatter and (b) Bland–Altman plots of the smartphone SDMT (1-week averages) and clinical SDMT scores. In the Bland-Altman plots, the solid lines represent the mean difference, and the dashed lines represent the 95% limits of agreement. Abbreviations: cSDMT, clinical Symbol Digit Modalities Test; sSDMT_M_, smartphone SDMT morning score; sSDMT_E_, smartphone SDMT evening score.

Correlation coefficients between the sSDMT and clinical measures are shown in Table 3. A strong correlation was found between the sSDMT and clinical SDMT (see also Figure 4(a)). Moderate correlations were found between the sSDMT and CVLT, BVMT-R and EDSS. For the concurrent validity, no large differences were found between the morning and evening scores or between different test periods (i.e. single, 1- or 2-week mean scores). Figure 4(b) shows the scatter and Bland–Altman plots between the 1-week averaged smartphone and clinical SDMT scores. On average, the morning and evening sSDMT scores were 8.9 and 9.5 points, respectively, lower than the clinical SDMT. This systematic bias (paired differences) was evenly scattered across the mean values. The limit of agreement was ± 15.0 and ± 14.9 points for the morning and evening sSDMT, respectively. Regression analyses show that a 1-point change in sSDMT score corresponds with 0.87 point change in the clinical SDMT across the patients and was not significantly confounded by age, sex, education level, arm function, severity of disability or smartphone size, see Table 4.

**Table 3.** Correlation coefficients between clinical tests and smartphone SDMT.

	Tests, *n* (mean ± SD)	SDMT		CVLT		BVMT-R		EDSS	
		*r*	*n*	*r*	*n*	*r*	*n*	ρ	*n*
Smartphone SDMT_M_									
Single score	1.0 ± 0.0	0.687	91	0.485	90	0.556	90	−0.478	91
1-week mean	3.6 ± 1.6	0.685	89	0.500	88	0.547	88	−0.484	89
2-week mean	5.8 ± 2.8	0.677	91	0.492	90	0.538	90	−0.494	91
Smartphone SDMT_E_									
Single score	1.0 ± 0.0	0.622	92	0.451	91	0.532	91	−0.416	92
1-week mean	3.4 ± 1.3	0.688	90	0.495	89	0.586	89	−0.458	90
2-week mean	5.6 ± 2.3	0.690	92	0.516	91	0.599	91	−0.480	92

SDMT: Symbol Digit Modalities Test; CVLT: California Verbal Learning Test; BVMT-R: Brief Visuospatial Memory Test-Revised; EDSS: Expanded Disability Status Scale; SDMT_M_: smartphone SDMT morning score; SDMT_E_: smartphone SDMT evening score.

All correlations were statistically significant (*p* < 0.001).

**Table 4.** Covariate analysis in the relation between smartphone and clinical SDMT.

	Clinical SDMT	*SE*	Change^a^ (%)
	*B* (95% CI)		
Smartphone SDMT			
Raw model	0.870 (0.679–1.062)	0.096	
Model adjusted for:			
Age	0.867 (0.616–1.119)	0.127	−0.3
Sex	0.896 (0.694–1.098)	0.102	3.0
Level of education	0.839 (0.647–1.031)	0.097	−3.6
AMSQ	0.905 (0.705–1.106)	0.044	4.0
NHPT-D	0.895 (0.669–1.121)	0.114	2.9
EDSS	0.888 (0.675–1.101)	0.107	2.1
Smartphone screen size			
Height	0.866 (0.672–1.059)	0.097	−0.5
Width	0.864 (0.669–1.059)	0.098	−0.7

SDMT: Symbol Digit Modalities Test; B: unstandardised regression coefficient; AMSQ: Arm function in Multiple Sclerosis Questionnaire; NHPT-D: Nine-Hole Peg Test Dominant hand; EDSS: Expanded Disability Status Scale.

a Covariate is considered a relevant confounder if the regression coefficient B changes more than 10%.

## Discussion

In this study, we investigated a self-administered smartphone-based SDMT on clinimetric properties to provide a basis towards clinical implementation. Within a 4-week period, an average increase in four points was observed, most likely attributable to practice effects. No large differences were found between sSDMT scores performed during the morning or evening, or whether single, 1- or 2-week averaged scores were used. The reliability estimates were highest for the 1- or 2-week mean scores, with corresponding SDC values of approximately 7 points; a score change of 7-points or more on the sSDMT can reliably be distinguished from measurement error. Construct validity was found for the sSDMT with median scores being significantly different between CI and CP patients. sSDMT scores were also different between patients with MS and HC, and even between CP patients and HC. Concurrent validity was established for the sSDMT in assessing information processing speed as it was strongly correlated with the clinical SDMT. The relation was not significantly confounded by age, sex, level of education, arm function, severity of disability or smartphone size. The sSDMT demonstrated moderate construct validity in assessing verbal memory, visuospatial memory and overall disability due to MS.

Earlier studies have examined the use of a self-administered SDMT assessed on the iPad in the clinic. One of these found a high correlation (ICC = 0.79) between the iPad and the written SDMT in 234 HC.^9^ An iPad-based SDMT investigated in patients with MS found high test–retest reliability (concordance correlation coefficient = 0.848) and strong correlation (*r* = 0.748) with the oral SDMT.^7^ A third iPad-based SDMT reported a Spearman’s correlation of 0.66.^10^ Our study found similar reliability to the second study, but lower correlation coefficients than the aforementioned studies. The lower correlations between the smartphone and clinical SDMT could be explained due to the iPad-based assessment was performed in the clinical setting and on the same day, whereas the sSDMT in our study was assessed in the days following the clinical visit and in the patients’ own environment. The assessment from the comfort of one’s own home may also be accompanied with more distractions during testing compared to in-clinic testing.

More similar to our study and more recently, smartphone-based SDMT applications have been investigated. A study with a composite smartphone assessment of information processing speed together with walking, manual dexterity and low-contrast visual acuity found high reliability (ICC = 0.90) and distinguished 69 HC and 116 patients with MS with an area under the curve (AUC) value of 0.92.^12^ In our current study, we found an AUC-value of 0.713 where only the SDMT was used. An interim analysis reported a Spearman’s correlation coefficient of 0.615 in 58 patients with MS at baseline using the average score of 1 week, and a moderate correlation with the psychological component of the Multiple Sclerosis Impact Scale-29.^8^ Finally, a previous report with the MS sherpa, the sSDMT was found to have a correlation of 0.784 with the cSDMT and a test–retest reliability of 0.874 in 25 patients with MS.^13^ Compared to this previous study, the MS sherpa sSDMT used in this study had a change in layout colour and the duration of the optional practice items as part of the instructions was reduced to a maximum of 15 seconds.

Altogether, currently available reports on iPad-based or smartphone-based SDMT support the reliability and validity found in our current study. None of the previous studies, however, investigated these clinimetric properties with regard to optimal frequency or time of the day of assessment. And more importantly, to our best knowledge, all previous reports on smartphone-based SDMT applications were performed on a standard and/or preconfigured smartphone provided by the study, whereas the MS sherpa sSDMT was performed on the participants’ own smartphone.

Limitations to be considered are the relatively short follow-up time and that the current study is a single-centre study. In addition, the current analyses did not include MRI metrics for MS disease activity or progression to relate to the smartphone-based assessment of cognitive function. Another limitation is the occurrence of a software bug that resulted in the removal of 11 patients from the analysis, this should prompt continuous alertness for occurrences of technical issues in future uses of technology-based biomarkers. Our results indicate that a score change of 7 points or more on the MS sherpa sSDMT can be clinically interpreted as a change outside of measurement error. However, we have yet to analyse the responsiveness of the sSDMT, that is, which amount of change can be considered *clinically relevant*. Finally, we have not accounted for practice effects of the repeated SDMT assessment. Indeed, the weekly averaged sSDMT scores were found to increase by at most 4-points at the end of follow-up compared to baseline. However, for both the reliability and validity analyses, no large differences were observed between single, 1- and 2-week averaged SDMT scores, implying no large influence of practice effects on the reliability and validity results.

## Conclusion

The self-administered smartphone-based SDMT was found to be reliable and had an SDC of 7 points. Group differences between HC and patients with MS, who were CP and CI, indicated construct validity for the sSDMT. The sSDMT had sufficient concurrent validity for assessment of information processing speed and was independent of potential confounders analysed within the study. Over a 4-week period, small practice effects were observed. No large differences in reliability or validity were observed between morning and evening assessment, or between single and weekly averaged sSDMT scores. Therefore, the sSDMT can be used to assess information processing speed remotely and more frequently in patients with MS.
